# 314. Using CRISPR/Cas9 to dissect the regions of MLL/AF9 that are critical for myeloid immortalization

**DOI:** 10.1093/ofid/ofad500.385

**Published:** 2023-11-27

**Authors:** Emma Yvanovich, Kyle Timmer, Michael Mansour, David B Sykes

**Affiliations:** Massachusetts General Hospital, Cambridge, Massachusetts; Massachusetts General Hospital, Cambridge, Massachusetts; Massachusetts General Hospital, Cambridge, Massachusetts; MGH, Boston, Massachusetts

## Abstract

**Background:**

High volumes of neutrophils are required for the successful granulocyte transfusion. Our goal is to expand neutrophil progenitors *ex vivo* to provide white blood cell transfusions of sufficient cell number. Creating a conditionally immortalized granulocyte-monocyte progenitor (GMP) would allow for exploration into the infinite expansion of GMPs to make a large volume of neutrophils.

The MLL/AF9 fusion oncoprotein is associated with mixed lineage leukemia (MLL), a form of acute myeloid leukemia (AML). MLL1 is a large gene with multiple binding sites associated with the development of MLL when broken and bound to a fusion parnter like AF9, highly efficient at causing MLL. We would like to understand whether certain regions of the gene might provide the capacity for ex vivo expansion without resulting in leukemia.

**Methods:**

To determine the most relevant portion of the MLL/AF9 gene, we conducted a "walkdown" with a custom CRISPR/Cas9 gRNA library to identify elements crucial for immortalization. The Cas9 and gRNAs will be delivered by lentiviral transduction. This walkdown employs a custom gRNA library with guides targeting each base pair along the ∼6000bp coding region. Transduction of MLL/AF9 dependent human leukemia cell lines THP1 and MOLM13 will be performed to identify those gRNA that target essential regions of MLL/AF9 in this drop-out screening approach.

**Results:**

Pilot experiments have been conducted to increase transduction efficiency for MLL/AF9 dependent cells. Transduction efficiency was maximized at 4 viral particles per cell in a 96 well plate (Figure 1). A larger scale walkdown pilot was conducted to maximize the amount of gDNA collected for PCR amplification. Sequencing of the PCR product will identify cells with essential areas knocked out (Figure 2).

Figure 1

**Figure:**
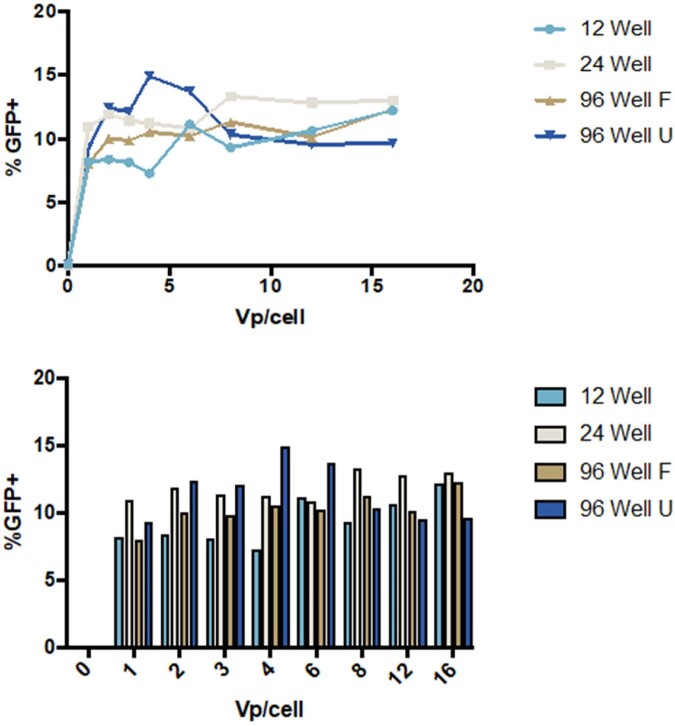

GFP positivity in transduced MLL/AF9 cells in differing plate sizes displayed in two formats.

Figure 2

**Figure:**
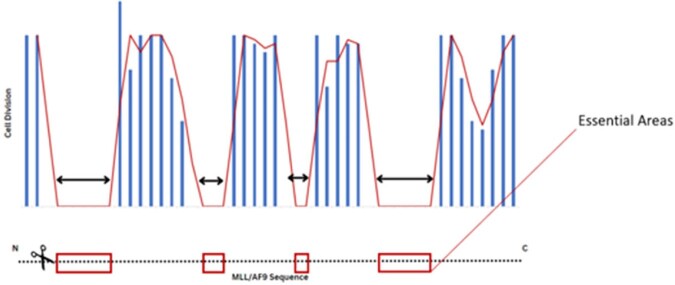

Predicted results of the walkdown experiment sequencing analysis.

**Conclusion:**

The association of the MLL/AF9 fusion oncogene with myeloid lineage leukemia offers a natural mechanism through which to study GMP immortalization. Specific areas of the coding region can be identified by conducted a "walkdown" experiment with CRISPR/Cas9 gRNA library to knock out each base pair of the oncogene. Regions of the gene can be identified as essential to survival when cells with these areas knocked out die off. Manipulation of these essential areas can be used for further study in the immortalization of GMPs.

**Disclosures:**

**Michael Mansour, MD, PhD**, Thermofisher: Grant/Research Support

